# Potential Probiotic Properties and Complete Genome Analysis of *Limosilactobacillus reuteri* LRA7 from Dogs

**DOI:** 10.3390/microorganisms12091811

**Published:** 2024-09-02

**Authors:** Yuanyuan Zhang, Mengdi Zhao, Yueyao Li, Shuang Liang, Xinkang Li, Yi Wu, Guangyu Li

**Affiliations:** 1 College of Animal Science and Technology, Qingdao Agricultural University, Qingdao 266109, China; 20222103021@stu.qau.edu.cn (Y.Z.); zmdi0808@126.com (M.Z.); liyueyao@stu.qau.edu.cn (Y.L.); 20232209008@stu.qau.edu.cn (S.L.); 20232209006@stu.qau.edu.cn (X.L.); 20232109046@stu.qau.edu.cn (Y.W.); 2 College of Animal Science and Technology, Jilin Agricultural University, Changchun 130118, China

**Keywords:** probiotics, *Limosilactobacillus reuteri*, dogs, probiotic properties, whole-genome sequencing

## Abstract

This study aimed to isolate and screen canine-derived probiotics with excellent probiotic properties. Strain characterization was conducted using a combination of in vitro and in vivo probiotic characterization and safety assessments, as well as complete genome analysis. The results showed that *Limosilactobacillus reuteri* LRA7 exhibited excellent bacteriostatic and antioxidant activities. The survival rate at pH 2.5 was 79.98%, and the viable counts after exposure to gastrointestinal fluid and 0.5% bile salts were 7.77 log CFU/mL and 5.29 log CFU/mL, respectively. The bacterium also exhibited high hydrophobicity, self-coagulation, and high temperature tolerance, was negative for hemolysis, and was sensitive to clindamycin. In vivo studies in mice showed that the serum superoxide dismutase activity level was 53.69 U/mL higher in the MR group of mice compared to that of the control group, the malondialdehyde content was 0.53 nmol/mL lower in the HR group, and the highest jejunal V/C value was 4.11 ± 1.05 in the HR group (*p* < 0.05). The *L. reuteri* LRA7 gene is 2.021 megabases in size, contains one chromosome and one plasmid, and is annotated with 1978 functional genes. In conclusion, *L. reuteri* LRA7 has good probiotic potential and is safe. It can be used as an ideal probiotic candidate strain of canine origin.

## 1. Introduction

The International Scientific Association for Probiotics and Prebiotics (ISAPP) defines probiotics as “live microorganisms which when administered in adequate amounts confer a health benefit on the host” [1]. Probiotics are only applicable to products that provide an appropriate number of viable bacteria (countries like Canada and Italy require a minimum viable cell count of 1 × 10^9^ CFU), complete genetic characteristics, and live microorganisms with clearly defined strains and that are safe and can bring health benefits to the host [1]. Lactic acid bacteria (LAB) are commonly used as probiotics, and *Limosilactobacillus reuteri* is a member of the genus Lactobacillus, which is considered to be a safe probiotic [2]. LAB have a variety of probiotic effects, among which reducing intestinal pathogenic bacteria to improve intestinal health is one of the important functions. It has been reported that LAB fermentation produces organic acids, bacteriocins, extracellular polysaccharides, hydrogen peroxide, and other antimicrobial substances. These can inhibit the growth of pathogenic bacteria by lowering the intestinal pH, disrupting the cell walls or cell membranes, and inhibiting intracellular gene and protein expression, thus improving the species composition of intestinal flora and maintaining intestinal homeostasis [3,4,5]. Mao et al. [6] found that the supplementation of *Lactobacillus acidophilus* in diets could increase the serum IgA and T-AOC levels, thereby improving the immune function and antioxidant capacity of dogs. Wang et al. [7] found that dietary supplementation with *Lactobacillus sake* can improve the composition of canine intestinal microbiota, enhance the antioxidant capacity, and promote metabolism. Another study indicates that complex lactic acid bacteria can alleviate the symptoms of diarrhea and vomiting in dogs by reducing the number of pathogenic bacteria in the canine gut [8]. Probiotics are widely used in pet diets due to their beneficial effects, which include improving nutrient digestibility; promoting animal growth; anti-inflammatory and anti-tumor properties; regulating immune function; enhancing the body’s antioxidant capacity; and improving the intestinal morphology and gut microbiota [9,10,11,12,13]. Furthermore, probiotics are considered a viable alternative to antibiotics because of their probiotic properties and characteristics, such as being free of side effects and residues.

Probiotics are widely used in all diets because of their beneficial probiotic properties. Within with the evolving role of pets, they have become our family members [14]. Pet health has become a primary concern for pet owners, leading to the emergence of a range of healthful pet foods, including probiotics [14]. Although most probiotics have been “Generally Recognised as Safe”, it is essential to assess and validate the safety of each strain [15]. The critical criteria for probiotic selection are, on the one hand, tolerance to the external environment as well as to the environment from the oral cavity to the gastrointestinal tract, e.g., gastric acid and bile salts, which is the first step for the strain to be able to survive in the host [16]. On the other hand, the adhesion and colonization of the strain to the host intestinal tissues are the key to the probiotic’s effect in the host and are among the compelling properties of probiotics [17]. In addition, spray drying is a commonly used method for the storage and transportation of probiotics, and probiotics with low thermal sensitivity have a lower survival rate during spray drying; thus, determining the thermal stability of probiotics is important for the industrial application of probiotics [18,19,20]. In the context of evolving food safety concepts, complete genome analysis, which enables the analysis of strain genetic information and risk assessment at the gene level, is widely used [21,22]. It has been reported that the use of host-derived probiotics is considered to be optimal due to the probiotic host–species specificity, which reduces the interference with the host’s own intestinal homeostasis [23,24]. However, there are few reports on evaluating probiotics of canine origin, and most commercial pet probiotic products contain strains of non-canine origin. The effectiveness of probiotic use varies. Therefore, there is a need for the screening and further evaluation of pet host-derived probiotics.

Given this, the study was conducted to isolate and characterize novel canine-derived probiotic strains from the feces of healthy Beagles, aiming to expand the database of canine-derived probiotics. The in vitro and in vivo evaluations of the probiotic properties and safety of these strains will provide a direction and a theoretical basis for their application in veterinary practice. Additionally, this research will offer guidance for incorporating these strains into pet food and serve as a reference for industrial production parameters, ultimately providing effective options for enhancing the health and welfare of dogs.

## 2. Materials and Methods

### 2.1. Bacterial Characteristics

#### 2.1.1. Isolation and Culture of Strains

Rectal feces was collected from 2 healthy adult beagles (Qingdao Agricultural University Experimental Base, Jimo, China) using the sterile swab method. The feces was transported to a laboratory in ice bath condition within 3 h. Then, 1 g of feces was taken and mixed with 9 mL deMan Rogosa and Sharpe (MRS) broth (Haibo, Qingdao, China), and then diluted to 10^−7^ in a gradient. Each gradient dilution was coated onto MRS agar plates and incubated at 37 °C for 24 h. After incubation, single colonies were picked, inoculated into MRS broth, and incubated at 37 °C for 24 h at 200 rpm/min. Strain purification was then performed by streaking on MRS agar medium. The strain purification process was repeated 5 times by picking single colonies, and then strain identification was performed until a single strain was obtained. The isolated strains were mixed in a 1:1 ratio (*v*/*v*) with 50% glycerol and stored at −80 °C.

#### 2.1.2. Identification of Strains

The isolated strains were initially identified through morphological observation and Gram staining. The physiological and biochemical properties of the strains were determined using bacterial microbiochemical tubes (HaiBo, China). At the same time, genomic DNA was extracted using the Bacterial Genome Extraction Kit (Solarbio, Beijing, China), and PCR amplification was performed with the 16S rRNA universal primers 27F (5′-AGAGTTTGATCCTGGCTCAG-3′) and 1492R (5′-TACGGCTACCTTGTTACGACTT-3′). The PCR products were subjected to agarose gel electrophoresis, and were then sent to Beijing Tsingke Biotech Ltd. (Beijing, China) for sequencing. The sequencing results were analyzed using BLAST on the NCBI website (available online: https://blast.ncbi.nlm.nih.gov/ (accessed on 23 September 2023) to compare homology. The comparison database was Core nucleotide, the program selected highly similar sequences, the comparison parameter E value was close to 0, the similarity value was >97%, and a phylogenetic tree was constructed using the Neighbor-Joining Method algorithm in MEGA 7 software.

### 2.2. In Vitro Studies

#### 2.2.1. First-Level Screening of Strains: Antimicrobial Activity

The Oxford Cup double-layer agar diffusion method was used to screen the LAB for inhibitory solid activity [25]. *E. coli* ATCC25922, *Salmonella* ATCC14028, and *Staphylococcus aureus* ATCC25923 were enriched and cultured with Tryptic Soy Broth (TSB) (HaiBo, China) medium as the indicator strains. Briefly, 10 mL of sterilized Tryptone Soy Agar (TSA) (Solarbio, China) was added as the bottom layer in a 9 cm sterile Petri dish. After solidifying, it was placed into an Oxford cup, and TSA containing 10^7^ CFU/mL of pathogenic bacteria was added as the second layer. After solidification, 200 μL of the fermentation broth from the isolated strain after 18 h of incubation was added to each well, and then incubated at 37 °C for 24 h. The diameter of the circle of inhibition was measured accurately at the end of incubation. A diameter of the inhibition circle <10 mm inicates no inhibitory activity, 10–16 mm indicates low inhibitory activity, 16–22 mm indicates medium inhibitory activity, and >22 mm indicates high inhibitory activity. The test was repeated 3 times, with 3 replicates each time.

#### 2.2.2. Secondary Screening of Strains: Antioxidant Activity

##### Cell-Free Supernatant Preparation

After the test strains were cultured at 37 °C for 18 h, they entered the growth stabilization period, and the absorbance value of OD600 nm reached 1.4, which was about 10^10^ CFU/mL. Then, 1 mL of bacterial suspension was sucked up and centrifuged at 4 °C at 10,000 rpm for 10 min. The supernatant was aspirated and filtered through a sterile 0.22 μm filter using an ultra-clean bench to obtain the cell-free supernatant (CFS).

##### 1,1-Diphenyl-2-Picryl-Hydrazyl (DPPH) Scavenging Capacity

The DPPH scavenging capacity of the strains was determined using the DPPH scavenging capacity kit (Grace, Suzhou, China). The optical density (OD) values were measured at 517 nm.

(1) $$DPPH\;radical\;scavenging\;rate=\left[1-\frac{{A_{Sample}}^{\;1}-{A_{Control}}^{\;1}}{{A_{Blank}}^{\;1}}\right]\times 100\%$$

${A_{Sample}}^{\;1}$ is the OD of a sample with DPPH working solution, ${A_{Control}}^{\;1}$ is the OD of a sample mixed with methanol, and ${A_{Blank}}^{\;1}$ is the OD of methanol with DPPH working solution.

##### 2,2′-Azinobis-(3-Ethylbenzthiazoline-6-Sulphonate) (ABTS) Scavenging Capacity

The ABTS scavenging capacity of the strains was determined using the ABTS scavenging capacity kit (Grace, China). The OD values were measured at 734 nm.

(2) $$ABTS\;radical\;scavenging\;rate=\left[1-\frac{{A_{Sample}}^{\;2}-{A_{Control}}^{\;2}}{{A_{Blank}}^{\;2}}\right]\times 100\%$$

${A_{Sample}}^{\;2}$ is the OD of a sample against the working solution, ${A_{Control}}^{\;2}$ is the OD of a sample with anhydrous ethanol, and ${A_{Blank}}^{\;2}$ is the OD of anhydrous ethanol vs. the working solution.

##### Superoxide Anion (O^2−^) Scavenging Capacity

The O^2−^ scavenging capacity was determined using the Superoxide Anion Scavenging Capacity Kit (Solarbio, China). The OD value was measured at 560 nm.

(3) $$O^{2-}\;radical\;scavenging\;rate\left(\%\right)=({A_{Blank}}^{\;3}-{A_{Sample}}^{\;3})/{A_{Blank}}^{\;3}\times 100\%$$

${A_{Sample}}^{\;3}$ is the OD of a sample and the working solution, and ${A_{Blank}}^{\;3}$ is the OD of distilled water and the working solution.

#### 2.2.3. Tertiary Screening of Strains: Acid Tolerance

The strains were incubated for 18 h. After centrifugation, the bacterial cell proteins were washed three times with PBS (pH 7.0), and then resuspended in an equal volume of PBS (pH 2.5) and incubated at 37 °C for 3 h [26]. The plate counting method was used to count the total number of colonies at 0 h and 3 h of incubation, and their survival rate was calculated.

(4) $$Surivability\left(\%\right)={N_{3h}}^{\;1}/{N_{0h}}^{\;1}\times 100\%\;$$

${N_{0h}}^{\;1}$ and ${N_{3h}}^{\;1}$ are the number of colonies (log CFU/mL) at 0 h and 3 h, respectively.

#### 2.2.4. Growth and Acid Production Capacity of Strain LRA7

The method was modified according to Zhang et al. [27]. The strains were cultured for 18 h, and then inoculated at 2% (*v*/*v*) in MRS broth for incubation. The OD_600_ nm values were measured at 0, 1, 2, 3, 6, 9, 12, 15, 18, 24, 30, 36, and 48 h, and the pH value of the culture solution was determined using a pH meter. To determine the growth of the strains in different pHs, they were inoculated into 2% (*v*/*v*) MRS broth at pH 1–7, and their OD600 nm values were measured at regular intervals.

#### 2.2.5. Gastrointestinal Tract Tolerance of Strain LRA7

After 18 h of incubation, the strain was centrifuged. The supernatant was discarded, and the bacterial somatic cells were washed three times with PBS (pH 7.0). Then equal volumes were resuspended in (0.1%, 0.3%, 0.5% *w/v*) bile salts (Bile salt content > 65%, Haibo, China) MRS broth, and artificial gastric (containing dilute hydrochloric acid and pepsin, pH = 2.0), intestinal (containing K_2_HPO_4_, NaOH, phosphate, trypsin, pH = 6.8), and gastrointestinal juices (Solarbio, China), respectively. Then, they were incubated at 37 °C for 4 h, 1.5 h, 3 h, and 4.5 h. Before and after incubation, 100 μL of the culture solution was collected and appropriately diluted for single colony counting using the plate counting method. The effective range for single colony counts was between 30 and 300.

#### 2.2.6. Surface Hydrophilicity of Strain LRA7

Hydrophobicity was determined using the method designed by Feng et al. [28]. The strain was cultured for 18 h, and then centrifuged to discard the supernatant. The bacterial somatic cells were washed three times with PBS (pH 7.0), and then resuspended in PBS (pH 7.0) and adjusted to an OD_600_ nm value of 0.25 ± 0.05 (A1). Then, the solution was thoroughly mixed with chloroform, xylene, and a 1:1 mixture of the two in equal volumes and incubated at 37 °C for 8 h. Subsequently, 200 μL of the aqueous phase was aspirated to measure the OD_600_ nm value (A_2_).

(5) $$Hydrophobicity\left(\%\right)=\left(1-\frac{A_{2}}{A_{1}}\right)\times 100\%$$

$A_{1}$ is the OD value before incubation, and $A_{2}$ is the OD value after 8 h of incubation.

#### 2.2.7. Auto-Aggregation Capacity of Strain LRA7

The method was slightly modified according to Ma et al. [29]. After 18 h of incubation, the strain was centrifuged, and the supernatant was discarded. The bacterial somatic cells were washed three times with PBS (pH 7.0), resuspended in an equal volume in PBS (pH 7.0), and then incubated at 37 °C without agitation. The absorbance values at OD_600_ nm were measured by aspirating 200 μL for 0 h and 8 h of incubation, respectively.

(6) $$Autoclustering\left(\%\right)=(1-\frac{A_{4}}{A_{3}})\times 100\%$$

$A_{3}$ is the OD value at 0 h of incubation, and $A_{4}$ is the OD value after 8 h of incubation.

#### 2.2.8. High-Temperature Resistance of Strain LRA7

The method was modified by Min et al. [30]. After 18 h of incubation, the cultures were heat-treated in a water bath at 37 °C, 50 °C, 60 °C, 70 °C, 80 °C, and 90 °C for 5 min, and then rapidly cooled on ice for about 2–5 min to bring the temperature of the bacterial solution down to room temperature. After appropriate dilution, 100 μL was spread onto MRS agar, and the number of colonies was counted after 24 h of inverted incubation at 37 °C.

#### 2.2.9. Characterization of Antimicrobial Substances of Strain LRA7

The determination of bacteriostatic substances was carried out using agar diffusion methods [31]. The fermentation supernatant was prepared using the previously described method, and then set aside. Bacteriocins were detected by subjecting the supernatant to a heat treatment at 100 °C for 10 min. Organic acids were identified by adjusting the pH of the supernatant to 7.0 using 1 mol/L NaOH. Hydrogen peroxide was determined by treating the supernatant with 0.5 mg/mL of catalase. The remaining steps of the procedure were identical to those for determining bacteriostatic activity.

#### 2.2.10. Antibiotic Sensitivity of Strain LRA7

The antibiotic susceptibility of *L. reuteri* LRA7 was tested using antibiotic sensitization tablets (Bio-knot, Wenzhou, China) according to the method described by Ahire et al. [32].

#### 2.2.11. Hemolytic Activity of Strain LRA7

After 18 h of incubation, the bacterial solution was dipped with an inoculating loop delineated on blood agar plates (containing 5% sheep blood) and placed at 37 °C for 48 h [33]. The hemolytic phenotype around the colonies was observed. *Staphylococcus aureus* ATCC25923 was used as a positive control.

### 2.3. In Vivo Studies

#### 2.3.1. Test Animals and Management

All the experimental animals were performed in accordance with the relevant guidelines and regulations and were approved by the Experimental Animal Ethics Committee of Qingdao Agricultural University (Approval No. DWKJ2024011201; Qingdao, China). Four-week-old Kunming mice obtained from Jinan Pengyue Experimental Animal Breeding Co., Ltd. (Jinan, China) were randomly divided into four groups, each consisting of 20 mice, with an equal number of males and females in each group. The mice were kept in a quiet environment at 25 °C for 12 h in light and 12 h in dark. The control group (CON) was supplemented with 0.2 mL of saline, and the experimental group was supplemented with 2 × 10^8^ CFU/mL (LR), 2 × 10^9^ CFU/mL (MR), and 2 × 10^10^ CFU/mL (HR) of the strain LRA7 culture, respectively. The mice were acclimatized for 7 d, and the test period lasted 28 d. The mice had access to food and water throughout the process. The mice were observed daily, and their body weight and food intake were recorded.

#### 2.3.2. Sample Collection

The mice were fasted for 12 h on the 28th day of the experiment. Subsequently, the mice were anesthetized with 0.3% sodium pentobarbital (0.15 mL/10 g), and then euthanized painlessly by cervical dislocation. Blood was collected from the ocular vein, centrifuged to obtain serum, and stored at −80 °C until analysis. After blood collection was completed, the mice were euthanized, and their organs were dissected and examined for health; the organs were collected and weighed to calculate the organ index [34]. Mouse jejunum and ileum tissues were also collected and fixed in 4% formaldehyde, and then embedded in paraffin and stained with hematoxylin–eosin [35]. The sections were observed and photographed using a light microscope, the length of the villi and the depth of the crypts were measured, and the ratio of the two (*V*/*C*) was calculated using ImageJ (2015) software [34].

### 2.4. Genome Analysis

The genomic DNA of strain LRA7 was extracted using a bacterial DNA extraction kit (TIANGEN, Beijing, China) [36]. Complete genome analysis was conducted using Illumina NovaSeq 6000 (Illumina, San Diego, CA, USA) and the PromethION platform (Oxford Nanopore Technologies, Oxford, UK). Coding genes were predicted from the assembled genome using Prokka software (version: 1.14.6) [37]. CRISPR prediction of the genome was performed using MinCED (version: 0.4.2). Functional gene comparison and annotation were conducted using UniProt (Version: UniProt 2022-03-09), Nr (Version: 2022-03-09), RefSeq (Version: 2022-03-09), GO (Version: 2022-03-09), COG (Version: 2022-03-09), KEGG (Version: 2021-05-03), and other databases [38,39,40,41]. Carbohydrase annotation, virulence factors, transporter proteins, and resistance genes were analyzed based on databases, such as CAZy (Version: 2021-09-24), VFDB (Version: 2022-03-11), TCDB (Version: 2022-03-11), and CARD (Version: 3.2.0) [42,43,44]. Genomic circle mapping was conducted using the R package (Version: 0.4.16).

### 2.5. Statistical Analysis

Each experiment was performed in triplicate, and the results were presented as the mean ± standard deviation. Statistical analysis was conducted using SPSS (version 26), and Duncan’s test was employed to analysis variance with statistical significance at *p* < 0.05. Figures were created using GraphPad Prism (version 8).

## 3. Results

### 3.1. LAB Isolation and Characterization

A total of 21 strains of *L. reuteri* were isolated by 16S rRNA sequence comparison, and the evolutionary tree is shown in Supplementary Figure S1.

### 3.2. In Vitro Results

#### 3.2.1. Strain Screening

The isolated strains showed different inhibitory activity degrees against the three pathogenic strains (Table 1). Among them, the strains whose circles of inhibition against the indicator strains were all greater than 16 mm in diameter were LR67, LRA1, LRA10, LRA6, and LRA7.

**Figure 1:**
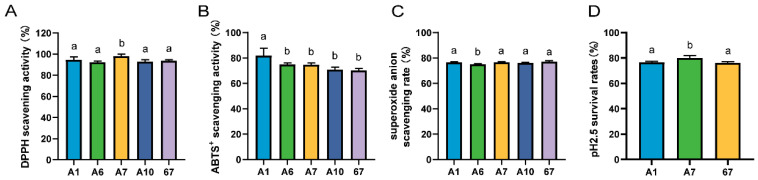
Antioxidant and acid tolerance results of the strain. (**A**) DPPH scavenging capacity. (**B**) ABTS scavenging capacity. (**C**) Superoxide anion scavenging capacity. (**D**) Acid tolerance. (a,b) Values with no letter or the same letter superscripts mean no significant difference (*p* > 0.05), while with different small letter superscripts mean significant difference (*p* < 0.05).

The five strains with vigorous bacteriostatic activity were then screened for antioxidant capacity. The scavenging rate of DPPH by LRA7 was 98.15 ± 1.90%, which was significantly higher than that of the other strains (Figure 1A). The best ABTS scavenging capacity was LRA1 at 81.75 ± 6.03% (Figure 1B), and for the scavenging of the superoxide anion, the lowest scavenging capacity was found in LRA6 (Figure 1C). The top three strains for antioxidant capacity were LRA1, LRA7, and LR67.

The strains screened for antioxidant capacity were further screened for acid tolerance. The results are shown in Figure 1D, and the survival rate of strain LRA7 at pH 2.5 was 79.98 ± 1.83%, which was significantly higher than that of LRA1 and LR67. Therefore, LRA7 continued to be evaluated as a candidate probiotic strain through tertiary screening.

#### 3.2.2. Morphological Characteristics of Strain LRA7

**Figure 2:**
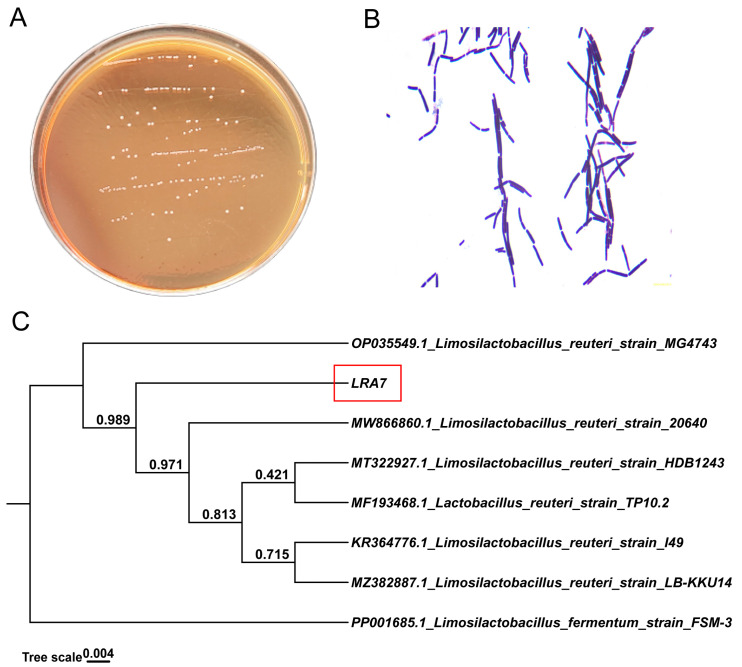
Colony morphology, cellular morphology, and evolutionary tree of strain LRA7. (**A**) Colony morphology of strain LRA7 on MRS agar. (**B**) Gram staining results of strain LRA7. (**C**) Evolutionary tree of strain LRA7.

Strain LRA7 is a round, creamy white colony with a smooth surface (Figure 2A). The microscopic examination of strain LRA7 shows that it is a rod-shaped gram-positive bacterium without bud cells and flagella (Figure 2B). In addition, the evolutionary tree (Figure 2C) shows that strain LRA7 is more phylogenetically similar to *L. reuteri* MG4743, with a bootstrap value greater than 98%.

#### 3.2.3. Growth Characteristics of Strain LRA7

**Figure 3:**
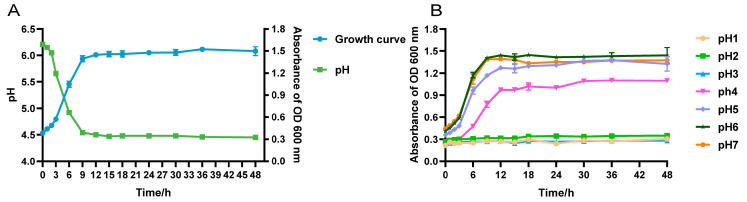
Growth capacity of strain LRA7. (**A**) Growth and acid production curves of strain LRA7. (**B**) Growth curve of strain LRA7 at pH 1–7.

Strain LRA7 grew slowly from 0 to 3 h, proliferated from 3 to 12 h, and was in the logarithmic growth phase. It entered the growth stabilization phase after 12 h. The pH of strain LRA7 increased from 0–3 h to 3–12 h, and then stabilized. Similarly, the decreasing trend of the pH was similar, with a slight decrease from 0 to 3 h, and a rapid decrease from 3 to 12 h, which finally stabilized at around 4.4 (Figure 3A). In addition, the study of the growth of the strains in different pH environments revealed that the growth of the strains could be wholly inhibited at pH ≤ 3, and at pH ≥ 4, the strains showed growth, but the optimal pH for growth was pH 6 (Figure 3B).

In addition, *L. reuteri* LRA7 produced physiological and biochemical results, as shown in Table 2. Strain LRA7 can utilize monosaccharides, such as galactose, glucose, arabinose, and xylose; disaccharides, such as sucrose, maltose, and lactose; and oligosaccharides such as raffinose.

#### 3.2.4. Analysis of Gastrointestinal Tolerance

**Figure 4:**
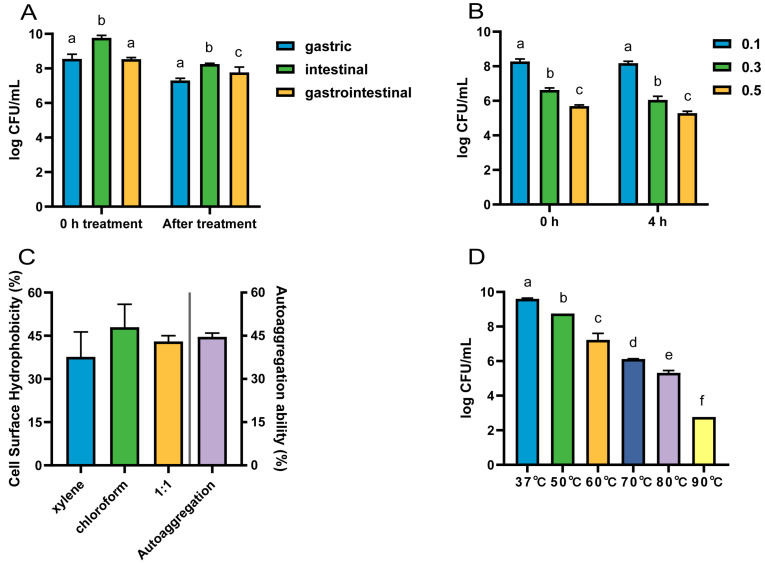
The tolerance of strain LRA7. (**A**) Gastrointestinal fluid tolerance. (**B**) Bile salt tolerance. (**C**) Hydrophobicity and self-condensation. (**D**) High-temperature resistance. (a–f) Values with no letter or the same letter superscripts mean no significant difference (*p* > 0.05), while with different small letter superscripts mean significant difference (*p* < 0.05).

The viable counts of strain LRA7 after the treatment with gastric, intestinal, and gastrointestinal fluids were 7.30 log CFU/mL, 8.26 log CFU/mL, and 7.77 log CFU/mL, respectively (Figure 4A). The survival count of LRA7 at 0.1% bile salt (8.18 log CFU/mL) was significantly higher than that at 0.3% (6.05 log CFU/mL) and 0.5% (5.29 log CFU/mL) bile salt survival concentrations (Figure 4B). The gastrointestinal environment is well tolerated by strain LRA7.

#### 3.2.5. Hydrophobicity and Self-Coalescence Analysis

*L. reuteri* LRA7 has good hydrophobicity and self-coagulation. Hydrophobicity after the treatment with xylene, chloroform, and an equal volume mixture of the two was 37.67%, 47.99%, and 43.02%, respectively, and the self-coagulation rate for 8 h was 44.62% (Figure 4C).

#### 3.2.6. High-Temperature Tolerance Analysis

The viable counts of *L. reuteri* LRA7 showed a gradual decrease with the increase in treatment temperature. However, the viable bacterial count remained up to 5.32 log CFU/mL after the heating treatment at 80 °C for 5 min (Figure 4D). Therefore, strain LRA7 has good high-temperature tolerance.

#### 3.2.7. Characterization of Bacteriostatic Substances

**Figure 5:**
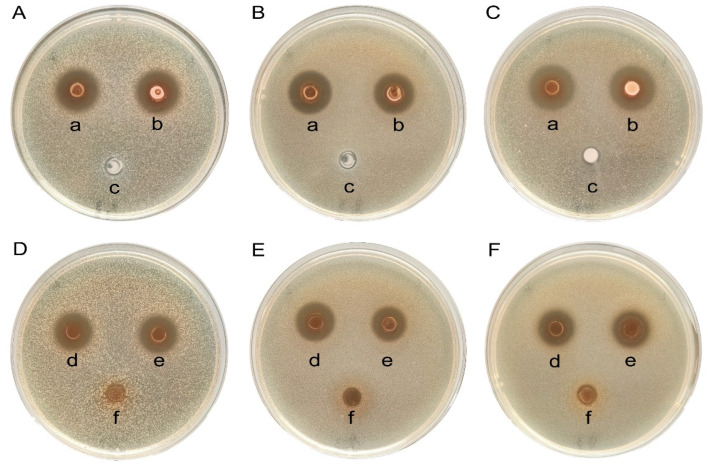
Characterization of bacteriostatic activity of strain LRA7. (**A** and **D**) *E. coli*. (**B** and **E**) *S. aureus*. (**C** and **F**) *Salmonella*. (a) Cell-free supernatant of strain LRA7. (b) Bacterial suspension of strain LRA7; (c) Bacterial body protein of strain LRA7. (d) Cell-free supernatant heat-treated at 100 °C. (e) Catalase-treated cell-free supernatant. (f) pH-neutralized cell-free supernatant (**D**–**F**).

From Figure 5A–C, it can be seen that the bacterial suspension and the CFS had an inhibitory effect, while the bacterial body protein had no inhibitory effect. Therefore, substances with bacteriostatic activity may exist in the CFS. Then, the identification of possible bacteriostatic substances in the CFS revealed that after the inactivation of bacteriocins by heating at 100 °C or the decomposition of hydrogen peroxide by catalase, the CFS still had a bacteriostatic effect (Figure 5 (d,e)). In contrast, there was no bacteriostatic effect after neutralizing the CFS (pH adjusted to 7.0) (Figure 5 (f)). This indicates that the organic acid is responsible for the bacteriostatic effect of strain LRA7.

#### 3.2.8. Antibiotic Sensitivity

The results of the drug sensitivity test of *L. reuteri* LRA7 are shown in Table 3. Strain LRA7 was susceptible to the β-lactams antibiotics and broad-spectrum antibiotics, but resistant to oxacillin and cotrimoxazole. It was resistant to the aminoglycoside antibiotics and the fluoroquinolone antibiotics.

#### 3.2.9. Hemolytic Activity

*L. reuteri* LRA7 was not hemolytic (Supplementary Figure S2a), but the positive control Staphylococcus aureus was β-hemolytic (Supplementary Figure S2b).

### 3.3. In Vivo Safety Assessment in Mice

**Figure 6:**
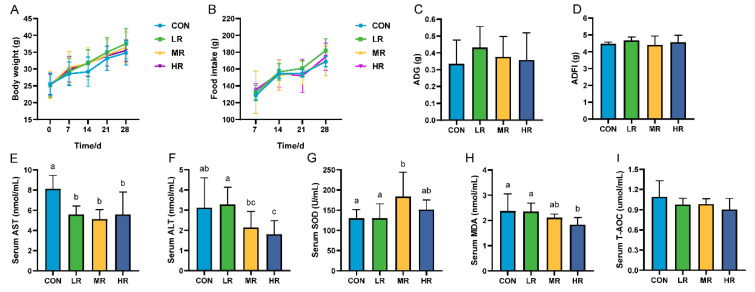
Effect of LRA7 on growth, feed intake, and serum biochemical indices in mice. (**A**) Mouse body weight. (**B**) Mouse feed intake. (**C**) Average daily gain. (**D**) Average daily food intake. (**E**) Aspartate aminotransferase. (**F**) Alanine aminotransferase. (**G**) Superoxide dismutase (**H**) Malondialdehyde. (**I**) Total antioxidant capacity. (a–c) Values with no letter or the same letter superscripts mean no significant difference (*p* > 0.05), while with different small letter superscripts mean significant difference (*p* < 0.05).

This study showed no significant difference in body weight, feed intake (Figure 6A–D), and organ index (Supplementary Figure S3) among the mice groups. Regarding the serum biochemical findings, the aspartate aminotransferase (AST) levels were significantly higher in the control mice than those in the experimental group. The alanine aminotransferase (ALT) levels were significantly higher in the CON group than those in the HR group, but were not significantly different from those in the LR and MR groups (Figure 6E,F). Regarding the antioxidants, the superoxide dismutase (SOD) levels were significantly higher in the MR group than those in the CON and LR groups. The serum malondialdehyde (MDA) levels were significantly lower in the HR group than those in the CON and LR groups, and there was no significant difference in the total antioxidant capacity (T-AOC) levels between the groups (Figure 6G–I).

**Figure 7:**
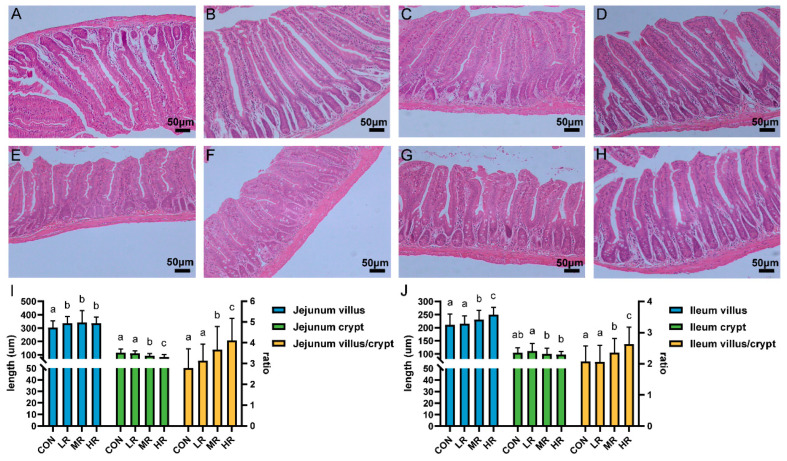
Effect of strain LRA7 on intestinal morphology of the jejunums and ilea in mice. (**A**) Jejunum of mouse in the CON group. (**B**) Jejunum of mouse in the LR group. (**C**) Jejunum of mouse in the MR group. (**D**) Jejunum of mouse in the HR group. (**E**) Ileum of mouse in the CON group. (**F**) Ileum of mouse in the LR group. (**G**) Ileum of mouse in the MR group. (**H**) Ileum of mouse in the HR group. (**I**) Jejunal villus length, crypt depth, and V/C values. (**J**) Ileal villus length, crypt depth, and V/C values. (a–c) Values with no letter or the same letter superscripts mean no significant difference (*p* > 0.05), while with different small letter superscripts mean significant difference (*p* < 0.05).

The sections of the jejunums and ilea of all the mice showed intestinal tissues that were structurally intact without apparent lesions (Figure 7A–H). The results of measuring the villus length and the crypt depth showed that the villus length of the jejunum experimental group was significantly higher than that of the control group. The villi of the ilea in the mice MR and HR groups were significantly longer than those of the CON and LR groups, the crypt depth of the jejunums and ilea was significantly lower in the MR and HR groups than those of the CON and LR groups, and the V/C value of the mice in the MR and HR groups was significantly higher than those of the CON and LR groups (Figure 7I,J).

### 3.4. Analysis of the Complete Genome of L. reuteri LRA7

#### 3.4.1. General Characterization of the Genome of Strain LRA7

**Figure 8:**
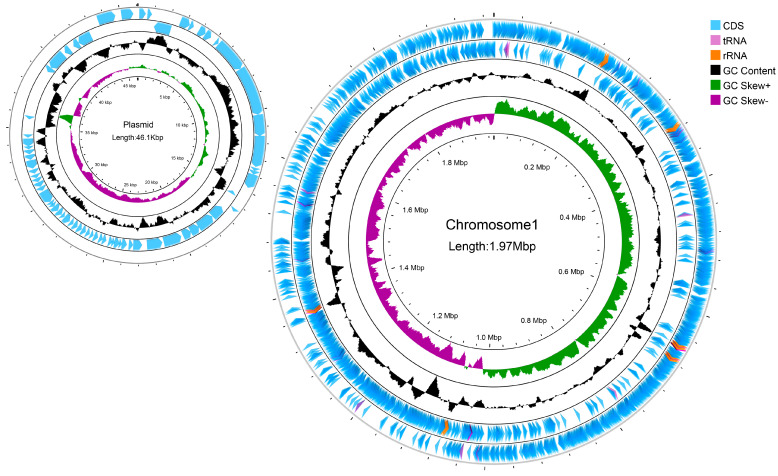
A genomic circle map of strain LRA7. In order from the outside to the inside, the first circle is the CDs, tRNAs, and rRNAs on the positive strand of the genomic sequence; the second circle is the CDs, tRNAs, and rRNAs on the negative strand of the genomic sequence; the third circle is the GC content curve of the genomic sequence; and the fourth circle is the GC skew curve of the genomic sequence, with green indicating that the G content is larger than the C content, and purple indicating that the G content is smaller than the C content.

The *L. reuteri* LRA7 genome is 2.021 M and consists of two loops (Figure 8). As shown in Table 4, one of them has a chromosome size of 1,974,833 bp, with a Guanine + Cytosine (G + C) content of 38.86%, and the other has a plasmid of 46,076 bp, with a G + C content of 34.33%, which predicted the number of tRNA and rRNA genes of 69 and 18. In addition, there are two hundred and nineteen pseudogenes, two CRISPRs, eight gene islands, one of which was a metabolic island, and eight prophages (Supplement Tables S1–S3). Some tolerance-related genes and virulence factors are shown in Supplement Tables S4 and S5.

#### 3.4.2. Functional Annotation of Strain LRA7 Genome

**Figure 9:**
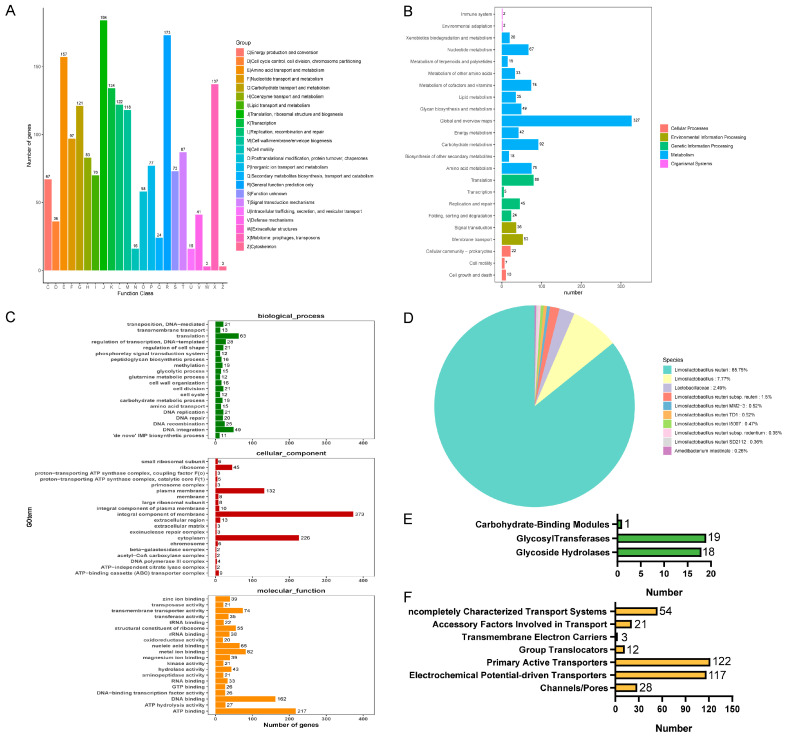
Whole-genome sequencing of strain LRA7. (**A**) Cluster of Orthologous Groups of proteins result classification chart. (**B**) Kyoto Encyclopedia of Genes and Genomes pathway result classification chart. (**C**) Gene Ontology-annotated gene enrichment maps for each secondary function. (**D**) Non-Redundant Protein Database species distribution map. (**E**) Carbohydrate-active enzyme annotation chart. (**F**) Transporter Classification Database transporter protein annotation map of LRA7 strain genome.

To obtain more comprehensive gene information, we performed gene function annotation. There were 1897 genes predicted in the COG database, which were categorized into 23 classes of immediate homologs, among which the top three in the number of annotated genes were translation, ribosomal structure, and biogenesis; general function prediction only; and amino acid transport and metabolism (Figure 9A). The use of the KEGG database predicted 1133 genes, most of which were related to global and overview maps, carbohydrate metabolism, and translation (Figure 9B). The annotation results of the GO database are shown in Figure 9C. The annotation results in the biological process were mainly related to translation, the cellular component was mainly related to an integral component of the membrane, and molecular function was mainly related to ATP binding. In NR database annotation, *L. reuteri* accounted for all the homologous sequence species of strain LRA7 by 97.25% (Figure 9D), which is consistent with the 16sRNA results. In addition, we annotated 38 carbohydrases in the CAZy database belonging to three subclasses, glycoside hydrolases, glycosyl transferases, and carbohydrate-binding modules (Figure 9E). Fifteen virulence factors were annotated in the VFDB (president more than 60%) (Table S5). Three hundred and fifty-seven membrane transporter protein genes were annotated in the TCDB database, of which the most commonly annotated were the primary active transporters (Figure 9F).

## 4. Discussion

The alarming increase in the number of antibiotic-resistant bacteria has led to the treatment of bacterial infections becoming less and less efficient, making it one of the biggest threats to global health [45]. Probiotics are considered to be a promising alternative to antibiotics [45]. In this study, we isolated and identified canine-derived LAB and combined in vitro probiotic evaluation with complete genome analysis to explain the probiotic function of *L. reuteri* and expand the database of canine-derived probiotics.

All the LAB isolated and characterized in this study showed bacteriostatic activity against the indicator strains. The broad-spectrum inhibitory activity of these strains was consistent with the inhibitory activity of LAB reported by Jose et al. [46]. Among the strains, LR67, LRA1, LRA10, LRA6, and LRA7 showed a moderate level of inhibitory activity against three pathogenic bacteria, which was superior to the other strains. The reason for the difference in bacteriostatic activity of the strains may be due to the different production of bacteriostatic active substances, or the different amounts of bacteriostatic substances produced by the different strains. It has been demonstrated that LAB can produce active antimicrobial substances, such as organic acids, hydrogen peroxide, and bacteriocins, to inhibit the growth of pathogenic bacteria [3]. Our further study of the strains with bacteriostatic activity revealed that CFS remained bacteriostatic after the heat treatment and the catalase treatment, while CFS lost its bacteriostatic activity after neutralizing the pH. Therefore, we hypothesized that the organic acids produced by strain LRA7 are the main antibacterial active substances. The results of this study align with those reported by Reuben et al., who found that organic acids are one of the active antibacterial substances produced by LAB [47]. However, the bacteriostatic effect of strain LRA7 differed from the bacteriostatic effect of LAB reported by Reuben et al [47]. The reason for this result may be due to the different sources of the strains or the different concentrations of the tested strains.

Oxidation is a necessary process for cellular metabolism in an organism. However, when the body is subjected to oxidative stress, the high concentration of reactive oxygen species generated can lead to aging and various chronic diseases [48]. LAB have recently been reported as a natural antioxidant [13,49]. The scavenging ability of *L. reuteri* LRA7 for DPPH (98.15%) and ABTS (74.61%) was significantly stronger than the scavenging ability of *L. reuteri PSC102* for DPPH (34.31%) and ABTS (17.84%), as reported by Ali et al. [50]. Probiotic supplementation has been reported to improve the body’s antioxidant capacity by increasing its antioxidant enzymes [51]. Therefore, we hypothesized that the high antioxidant activity of strain LRA7 may result from its higher capacity to produce antioxidant enzymes. In the present study, the supplementation of mice with *L. reuteri* LRA7 increased the level of serum SOD. It decreased the level of serum MDA, which is consistent with the results of Chai et al. [13], who supplemented *L. reuteri* in chicken diets. In addition, thioredoxin trxA, hydroxymethylglutaryl-CoA reductase, thioredoxin reductase, and thiol-disulfide isomerase or thioredoxin were also annotated in the whole genome of the strain LRA7 antioxidant genes. Thus, *L. reuteri* LRA7 has a good antioxidant capacity. The strong antioxidant properties of strain LRA7 may hold promise for various applications, including slowing down pet aging, prolonging pets’ lifespan, and reducing pets’ stress.

The optimal growth pH of strain LRA7 was pH 6, and growth was inhibited at pH < 5, which is consistent with the growth environment of LAB isolated by Yang et al. [52]. Probiotic tolerance to the harsh environment of the gastrointestinal tract and colonization in the gastrointestinal tract are conducive to long-lasting probiotic effects in the body and are among the factors for probiotic selection [53]. This mainly includes resistance to a low pH in the stomach, high bile salt concentrations in the intestine, and various digestive enzymes in the gastrointestinal tract [17]. In this study, *L. reuteri* LRA7 was well tolerated in pH 2.5, 0.5% bile salts, and the gastrointestinal fluids. Moreover, the survival rate of the strain was higher than the reported tolerance of *L. reuteri* to acid and bile salts [54]. However, the tolerance to gastrointestinal fluids was not as different as that reported by Xu et al. [55], who stated that *L. reuteri* could proliferate in the gastrointestinal environment. This may be due to the different sources of the strains. The ability of probiotics to withstand the conditions of gastrointestinal fluids enables a greater number of live bacteria to reach the intestine, where they can exert their probiotic effects. Additionally, these strains can be supplemented through oral administration and other methods, simplifying clinical applications, while ensuring their effectiveness. Bacterial adhesion is influenced by changes in the expression levels of cell surface proteins and environmental influences on the expression of surface proteins that affect cell surface hydrophobicity and self-cohesion [56]. Hydrophobicity and self-coagulation are the key influencing factors for LAB colonization in the intestine [53]. The stronger hydrophobicity and higher self-coagulation rate of *L. reuteri* LRA7 help it colonize the intestine and perform its probiotic function better. In addition, the strain still had more than half of the surviving numbers after the 80 °C high-temperature treatment, and its high-temperature tolerance was significantly better than that of the pre-existing *L. reuteri I2*. This also provides more favorable conditions for the industrial application of strain LRA7.

Safety is another crucial factor to consider when selecting potential probiotics. Therefore, we performed an in vitro hemolysis test and an antibiotic susceptibility test as well as an in vivo safety assessment of the mice for the safety of strain LRA7. The results of the tests showed that *L. reuteri* LRA7 was not hemolytic. Regarding antibiotic resistance, it has been reported that most LAB are susceptible to β-lactams antibiotics and resistant to aminoglycosides antibiotics, which is consistent with the results of the present study [57,58]. The other studies have shown that antibiotic resistance only leads to risk when resistance can be transferred [59]. Although the lincosamide antibiotic resistance gene was predicted during genome-wide prediction, our drug sensitivity test results showed that the LRA7 strain was susceptible to clindamycin and not resistant. Therefore, it indicates that *L. reuteri* is safe regarding antibiotic resistance without the risk of a resistance gene-phenotype and transfer. In addition, we evaluated the in vivo safety of *L. reuteri* via a mouse gavage test. The study results showed no mortality in all the mice throughout the test period, and *L. reuteri LRA7* has no adverse effects on growth and organ development in the mice. In addition, it has been reported that probiotic supplementation improves animal nutrient absorption by increasing the villus length and decreasing the crypt depth [60]. In the present study, the sections of the jejunums and ilea of the mice showed intact intestinal tissue without lesions, and the V/C values of the mice in the MR and HR groups were significantly higher than those in the control group. It reveals that supplementation with *L. reuteri* LRA7 enhances nutrient absorption and improves the intestinal morphology in mice.

In order to gain a deeper understanding of the biological capabilities of *L. reuteri* LRA7, we performed complete genome analysis. The *L. reuteri* LRA7 genome is 2,020,909 bp in size, contains one chromosome and one plasmid, consists of 2126 genes, and the complete genome has a G + C content of 39.26%, which is similar to that reported for *L. reuteri* [61]. The number of genes in probiotic mRNA and tRNA has been reported to correlate with the better adaptation of the strain to the environment [62]. The numbers of mRNAs and tRNAs of strain LRA7 are higher than those reported for *L. reuteri*, so strain LRA7 may be better adapted to the environment [61,63]. This feature may be related to the fact that strain LRA7 can be well cultured in an in vitro environment and has better gastrointestinal environmental adaptation. The complete genome of *L. reuteri* LRA7 was annotated with 1978 functional genes. Firstly, a variety of stress-related protein genes were annotated (Table S4), such as peroxide stress protein *YaaA*, heat shock protein *HtpX*, *F0F1 ATP* synthase subunit delta, and *H*^+^/*Cl*^−^ *antiporter ClcA*. The stress-related proteins not only reveal genetic adaptation, but also regulate evolution resistance [64], which is associated with Lactobacillus royales bile salt tolerance, acid tolerance, high-temperature tolerance, and high adhesion probiotic properties. In addition, *L. reuteri* belongs to the functional LAB group, which can metabolize carbohydrates to produce lactic acid [64]. *L. reuteri* LRA7 has essential genes encoding carbohydrate metabolism, *FBA*, *PFK*, *PGI*, and *GAPDH*. These genes are involved in two glucose metabolism pathways, glycolysis and pentose phosphate, which conflict with only the *L. reuteri* pentose phosphate pathway, as Morita et al. reported [63]. The reason may be related to the different strain sources and the strain’s specificity. In addition, 38 carbohydrate-active enzyme genes were also annotated in the CAZy database, and many glycoside hydrolases and glycosyl transferases are involved in carbohydrate metabolism and transport. They have important roles in promoting material metabolism and improving nutrient digestibility in the body. In addition, we annotated 401 virulence factor-related genes in the VFDB database, with 15 genes having greater than 60% gene similarity (Table S5). It is worth noting that the virulence factors are divided into two categories: surface factors involved in host cell colonization and factors that cause damage to the host tissues [65]. A large proportion of virulence factors are not involved in pathogenicity, are essential for probiotics, and are considered health factors. A larger proportion of virulence genes may be pathogenic, and therefore present a greater risk. Consequently, the thorough safety assessment of these strains is essential before they can be utilized for clinical applications and industrial production. Virulence and resistance genes are still the center of debate on the safety of probiotics, and more in-depth studies are needed to elucidate their functional roles and their impact on the clinical application of probiotics.

## 5. Conclusions

In summary, a strain of *L. reuteri* LRA7 exhibiting strong probiotic properties was isolated from canine feces. The effective bacteriostatic activity of LRA7 demonstrated its significant bacteriostatic capability, while its strong free radical scavenging ability and oxidase production in mice indicated the strain’s antioxidant potential. In vitro probiotic characterization and complete genome analysis revealed that strain LRA7 has good tolerance and adaptability to the harsh environment of the gastrointestinal tract. This in vivo study demonstrated that supplementation with *L. reuteri* LRA7 had no toxic side effects in mice, increased the antioxidant capacity, and improved the intestinal morphology. The current study proves the safety of the strain. Consequently, strain LRA7 shows promise for a variety of applications, including reducing oxidative stress in the body, delaying aging, preventing bacterial diarrhea, and enhancing intestinal health. *L. reuteri* LRA7 could serve as an excellent candidate for a pet probiotic.

## Figures and Tables

**Table 1:** Results of bacteriostatic activity of 21 strains of *L. reuteri*.

Strains	*E. coli*	*Salmonella*	*S. aureus*
LR67	++	++	++
LRA1	++	++	++
LRA10	++	++	++
LRA6	++	++	++
LRA7	++	++	++
LR6	+	++	++
LR61	++	+	++
LRA15	+	++	++
LRA21	+	++	++
LR8	+	++	++
LR19	+	++	++
LR49	+	++	++
LRG4	+	++	++
LR2	+	++	++
LR1	+	++	++
LRG3	+	++	++
LR62	+	+	++
LRA3	+	++	++
LR14	+	++	++
LRG2	+	+	++
LRG1	+	++	++

Symbols <10 −; 10–16 +; 16–22 ++; >22 +++.

**Table 2:** Physiological and biochemical characterization of strain LRA7.

Items	Results	Items	Results
Glucose	+	Inulin	−
Cellobiose	+	Raffinose	+
Galactose	+	Hydrogen sulfide test	−
Sucrose	+	Sorbitol	−
Maltose	−	Aesculin	−
Mannitol	−	Hydrogen sulfide test	−
Xylose	+	Methyl Red test	−
Lactose	+	Voges–Proskauer test	−
L-Rhamnose	−	Gelatin liquidized test	−
Pectinose	+	Urease test	−
Salicin	−	Citrate test	−
Motility	−		

Symbols +: positive reaction; −: negative reaction.

**Table 3:** Antibiotic resistance of strain LRA7.

Antimicrobial Classes	Antimicrobial Agents	Disk Dose (μg)	Inhibition Zone Diameters/mm (IZD) ^a^		
			≤15 mm (R)	16–20 mm (I)	≥21 mm (S)
β-lactams antibiotics	Penicillin	10			23.17 ± 3.18 ^S^
	Oxacillin	1	X ^R^		
	Ampicillin	10		19.15 ± 1.53 ^I^	
	Piperacillin	100			25.00 ± 1.12 ^S^
	Imipenem	10			30.71 ± 2.83 ^S^
Aminoglycosides antibiotics	Streptomycin	10	X ^R^		
	Gentamicin	10	X ^R^		
	Amikacin	30	X ^R^		
	Kanamycin	30	X ^R^		
Broad-spectrum antibiotics	Tetracycline	30		16.32 ± 2.45 ^I^	
	Chloramphenicol	30			22.77 ± 0.68 ^S^
	Minocycline	30			22.70 ± 0.65 ^S^
	Doxycycline	30		20.74 ± 0.92 ^I^	
	Cotrimoxazole	25	X ^R^		
Macrolides	Azithromycin	15	X ^R^		
	Erythromycin	15	X ^R^		
	Clindamycin	2		16.27 ± 1.18 ^I^	
Fluoroquinolone antibiotics	Norfloxacin	10	X ^R^		
	Ciprofloxacin	5	X ^R^		
	Levofloxacin	5	X ^R^		

^a R^, resistant; ^I^, intermediate; ^S^, sensitive; X, no inhibition zone observed. Values are means, with SD of three replications.

**Table 4:** Features of *L. reuteri* LRA7 genome.

Attributes	Values
Chromosome size (bp)	1,974,833
Plasmid size (bp)	46,076
G + C content of chromosome (%)	38.86%
G + C content of plasmid (%)	34.33%
tRNA	69
rRNA	18
Pseudogene numbers	219
Pseudogene size (bp)	68,864
CRISPRs	2
Gene islands	4
Prophages	8

## Data Availability

The Illumina sequencing raw data were deposited in the NCBI database, Bio Project ID: PRJNA1068336.

## Supplementary Materials

The following supporting information can be downloaded at: https://www.mdpi.com/2076-2607/12/9/1811/s1. Table S1: CRISPR prediction of *Limosilactobacillus reuteri* LRA7. Table S2: Predicted results of the genomic islands to *Limosilactobacillus reuteri* LRA7. Table S3: Predicted results of the Prophage to *Limosilactobacillus reuteri* LRA7. Table S4: Tolerance related genes of *Limosilactobacillus reuteri* LRA7. Table S5: Prediction of virulence factor to *Limosilactobacillus reuteri* LRA7. Table S6: Prediction of resistance gene to *Limosilactobacillus reuteri* LRA7. Figure S1: Evolutionary tree of 21 strains of lactic acid bacteria. Figure S2: Hemolytic activity of *Limosilactobacillus reuteri* LRA7. (A) Face of blood plate; (B) Reverse of blood plate; (a) *Staphylococcus aureus* ATCC25923; (b) *Limosilactobacillus reuteri* LRA7. Figure S3: Effect of strain LRA7 on organ indices in mice. (A) heart coefficient; (B) liver coefficient; (C) spleen coefficient; (D) kidney coefficient; (E) thymus coefficient was calculated using organ weight/body weight.

## Abbreviations

LAB	Lactic acid bacteria
CFS	cell-free supernatant
DPPH	1,1-diphenyl-2-picryl-hydrazyl
MRS	Man, Rogosa, and Sharpe
OD	optical density
ABTS	2,2′-Azinobis-(3-ethylbenzthiazoline-6-sulphonate)
O^2−^	superoxide anion
TSB	Tryptic Soy Broth
TSA	Tryptone Soy Agar
GO	Gene Ontology
COG	Cluster of Orthologous Groups of proteins
VFDB	virulence Factors Database
KEGG	Kyoto Encyclopedia of Genes and Genomes
CAZy	Carbohydrate-Active enZYmes Database
TCDB	Transporter Classification Database
Nr	Non-Redundant Protein Database
CARD	Comprehensive Antibiotic Research Database
V/C	villus length/crypt depth
AST	aspartate aminotransferase
ALT	alanine aminotransferase
SOD	superoxide dismutase
MDA	malondialdehyde
T-AOC	total antioxidant capacity
G + C	Guanine + Cytosine
