# Supplementary material for: Potential Probiotic Properties and Complete Genome Analysis of Limosilactobacillus reuteri LRA7 from Dogs
Source: Microorganisms. 2024 Sep 2;12(9):1811. doi: 10.3390/microorganisms12091811 (PMC11605243; doi:10.3390/microorganisms12091811)
Supplement: Supplementary file 1 [file microorganisms-12-01811-s001.zip › microorganisms-3174890-supplementary.pdf]

Supplementary Material

Table S1. CRISPR prediction of *Limosilactobacillus reuteri* LRA7.

ID	start	end	score	crispr id	rpt unit seq
gnl|Bacteria|ctg_1	1152374	1153333	15	CRISPR1	GTTCTAAACATTATTGATTTGAAGTACATCTAAAAC
gnl|Bacteria|ctg_2	35467	35602	2	CRISPR2	TTTATTATATAGCCTTAAGTCTATATTGTCAAGAGGTAATTTT

Table S2. Predicted results of the genomic islands to *Limosilactobacillus reuteri LRA7*.

Gene ID	start	end	GI length	Product
-	1108869	1109678	809	hypothetical protein
-	1109675	1110748	1073	hypothetical protein
-	1110741	1111901	1160	hypothetical protein
rfbX_1	1111928	1113382	1454	Putative O-antigen transporter
-	1113404	1114390	986	hypothetical protein
-	1114413	1115675	1262	hypothetical protein
-	1115683	1116588	905	hypothetical protein
-	1116604	1117548	944	hypothetical protein

Table S3. Predicted results of the Prophage to *Limosilactobacillus reuteri* LRA7.

ID	PP start	PP end	attL sequence	attR sequence
Prophage 1	765713	766801	CAACAAGTAACG	CGTTACTTGTTG
Prophage 2	851176	852069	TGGCGTTCACGA	TGGCGTTCACGA
Prophage 3	1011933	1012220	TAAAAAAGATGTGCG	CGCACATCTTTTTTA
Prophage 4	1036306	1041059	TATTAGGAAAATGA	TCATTTTCCTAATA
Prophage 5	1330361	1363135	CATCGTCACTA	TAGTGACGATG
Prophage 6	1685622	1686830	TGACATCCCCA	TGGGGATGTCA
Prophage 7	2628	3221	TAACAAAAAATAGACATCTA	TAGATGTCTATTTTTTGTTA
Prophage 8	21395	22432	AAATACAGATTTTACA	TGTAAAATCTGTATTT

PP start: the starting position of the phage on the contig; PP end: the ending position of the phage on the contig; attL sequence: the sequence of the site to the left of the original phage; attR sequence: the site to the right of the original phage.

Table S4. Tolerance related genes of *Limosilactobacillus reuteri* LRA7.

Gene locus	Gene name	Gene function
**Universal stress family protein**		
ctg_00559	*YaaA*	peroxide stress protein
**Heat stress resistance**		
ctg_00823	*DnaK*	molecular chaperone DnaK
ctg_00824	*DnaJ*	molecular chaperone DnaJ
ctg_00822	*GrpE*	molecular chaperone GrpE
ctg_00821	*HrcA*	heat-inducible transcriptional repressor HrcA
ctg_00299	*hslO*	molecular chaperone Hsp33
ctg_01611	*ctsR*	transcriptional regulator of stress and heat shock response
ctg_00402	*GroES*	co-chaperone GroES
ctg_00403	*groEL, HSPD1*	chaperonin GroEL
ctg_00264	*HtpX*	heat shock protein
**Cold-shock stress resistance**		
ctg_01627	-	Cold-shock protein
ctg_00715	*CSD*	Cold-shock' DNA-binding domain
**Bile salt resistance**		
ctg_00842	-	bile salt hydrolase
ctg_00842	*CBAH*	choloylglycine hydrolase family
ctg_00898	*ppaC*	manganese-dependent inorganic pyrophosphatase
ctg_00530	-	F0F1 ATP synthase subunit delta
**pH stress resistance**		
ctg_01203	*Asp23*	alkaline shock protein (Asp23) family
ctg_01225	*Asp23*	alkaline shock protein (Asp23) family
ctg_00903	*Asp23*	alkaline shock protein (Asp23) family
ctg_00066	*ClcA*	H+/Cl- antiporter ClcA
ctg_00206	*ClcA*	H+/Cl- antiporter ClcA
ctg_01089	*ClcA*	H+/Cl- antiporter ClcA
ctg_00530	*atpH*	F-type H+-transporting ATPase subunit delta
ctg_00534	*atpC*	F-type H+-transporting ATPase subunit epsilon
ctg_00531	*atpA*	F0F1 ATP synthase subunit alpha
ctg_00533	*atpD*	F-type H+/Na+-transporting ATPase subunit beta
ctg_00532	*atpG*	F-type H+-transporting ATPase subunit gamma
ctg_00528	*atpE*	F-type H+-transporting ATPase subunit c
ctg_00527	*atpB*	F-type H+-transporting ATPase subunit a
ctg_00529	*atpF*	F-type H+-transporting ATPase subunit b
ctg_00174	*nhaC*	Na+:H+ antiporter, NhaC family
ctg_01940	*nhaC*	Na+:H+ antiporter, NhaC family
ctg_00174	*nhaC*	Na+:H+ antiporter, NhaC family
**Oxidative stress**		
ctg_00615	*trxA*	thioredoxin 1
ctg_01051	*fabI*	enoyl-[acyl-carrier protein] reductase I

ctg_00584	-	NADH oxidation
ctg_00172	-	Putative NADH-flavin reductase
ctg_00401	-	NADH/NAD ratio-sensing transcriptional regulator Rex
ctg_01165	*ahpC*	NADH-dependent peroxiredoxin subunit C
ctg_01955	-	NADHX epimerase activity
ctg_00227	*mvaA*	hydroxymethylglutaryl-CoA reductase
ctg_00427	*trxB,TRR*	thioredoxin reductase (NADPH)
ctg_01164	*trxB,TRR*	thioredoxin reductase (NADPH)
ctg_01296	-	Thiol-disulfide isomerase or thioredoxin
ctg_02030	*pcaC*	4-carboxymuconolactone decarboxylase
ctg_01780	-	NADPH-dependent 2,4-dienoyl-CoA reductase
ctg_00073	-	NADPH-dependent 2,4-dienoyl-CoA reductase
ctg_01058	*fabG*	OAR1`3-oxoacyl-[acyl-carrier protein] reductase
ctg_00584	-	NADH dehydrogenase

Table S5. Prediction of virulence factor to *Limosilactobacillus reuteri* LRA7.

VFDB internal ID	Gene	Virulence factor category
VFG006022	*rfbB*	Immune modulation
VFG005898	*rfbA*	Immune modulation
VFG006826	*lisR*	Regulation
VFG005582	*eno*	Exoenzyme
VFG005879	*hasC*	Immune modulation
VFG016490	*tuf*	Adherence
VFG000077	*clpP*	Stress survival
VFG012102	*groEL*	Adherence
VFG002182	*cpsI*	Immune modulation
VFG048851	*gndA*	Immune modulation
VFG045688	*cpsA/uppS*	Immune modulation
VFG037104	*msrA/B(pilB)*	Stress survival
VFG016424	*manA*	Immune modulation
VFG006089	*rfbD*	Immune modulation
VFG043573	*dnaK*	Adherence

Display only greater than 60% pident.

Table S6. Prediction of resistance gene to *Limosilactobacillus reuteri* LRA7.

Gene locus	Drug class	Resistance mechanism	AMR gene family
ctg_00931	lincosamide antibiotic	antibiotic inactivation	lincosamide nucleotidyltransferase (LNU)
ctg_00941	lincosamide antibiotic	antibiotic inactivation	lincosamide nucleotidyltransferase (LNU)

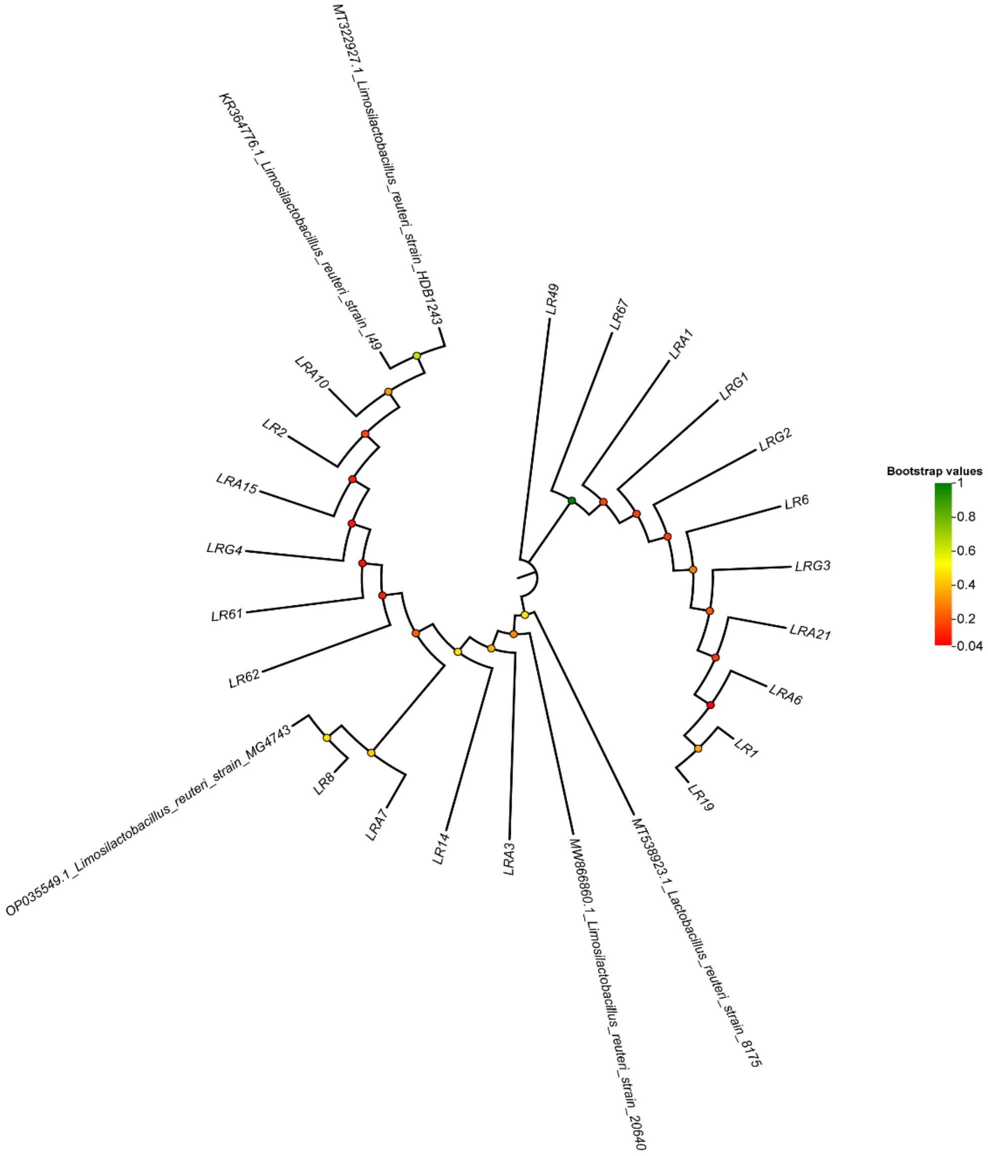

Figure S1. Evolutionary tree of 21 strains of lactic acid bacteria.

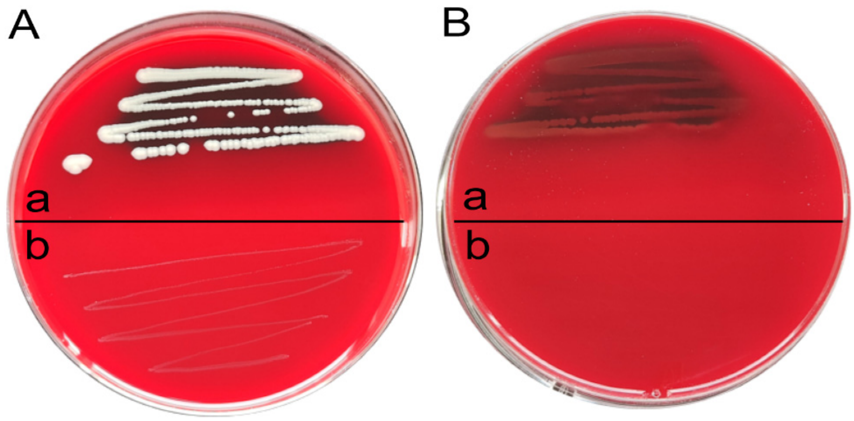

Figure S2. Hemolytic activity of *Limosilactobacillus reuteri LRA7*. (A) Face of blood plate; (B) Reverse of blood plate; (a) Staphylococcus aureus ATCC25923; (b) *Limosilactobacillus reuteri LRA7*.

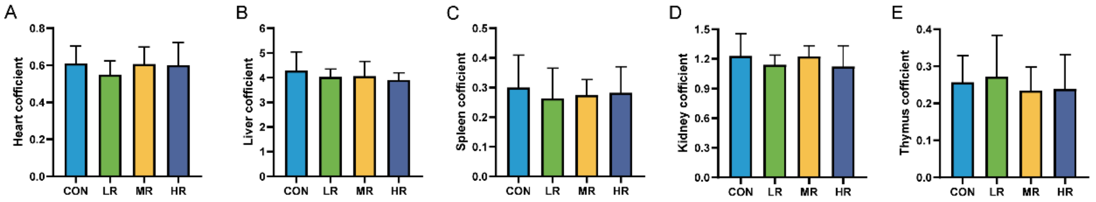

Figure S3. Effect of strain LRA7 on organ indices in mice. (A) heart coefficient; (B) liver coefficient; (C) spleen coefficient; (D) kidney coefficient; (E) thymus coefficient was calculated using organ weight/body weight.
